# High-harmonic generation from subwavelength silicon films

**DOI:** 10.1515/nanoph-2024-0468

**Published:** 2025-01-16

**Authors:** Kent Hallman, Sven Stengel, Wallace Jaffray, Federico Belli, Marcello Ferrera, Maria Antonietta Vincenti, Domenico de Ceglia, Yuri Kivshar, Neset Akozbek, Shroddha Mukhopadhyay, Jose Trull, Crina Cojocaru, Michael Scalora

**Affiliations:** PeopleTec, Inc. 4901-I Corporate Dr., Huntsville, AL 35805, USA; Institute of Photonics and Quantum Sciences Heriot-Watt University, SUPA Edinburgh, EH14 4AS, Edinburgh, UK; Department of Information Engineering, 9297 University of Brescia, 25123 Brescia, Italy; Nonlinear Physics Centre, Australian National University, Canberra, ACT 2601, Australia; US Army Space & Missile Defense Command, Tech Center, Redstone Arsenal, AL 35898, USA; Department of Physics, Universitat Politècnica de Catalunya, 08222 Terrassa, Barcelona, Spain; FCDD-AMT-MGR, DEVCOM AvMC, Charles M. Bowden Research Center, Redstone Arsenal, AL, 35898-5000, USA

**Keywords:** high-harmonic generation, nonlinear optics, Si nanophotonics, spatiotemporal dynamics, time varying media

## Abstract

Recent years have witnessed significant developments in the study of nonlinear properties of various materials at the nanoscale. Often, experimental results on harmonic generation are reported without the benefit of suitable theoretical models that allow assessment of conversion efficiencies compared to the material’s intrinsic properties. Here, we report experimental observations of *even and odd harmonics up to the 7*th, generated from a suspended subwavelength silicon film resonant in the UV range at 210 nm, the current limit of our detection system, using peak power densities of order 3 TW/cm^2^. We also highlight the time-varying properties of the dielectric function of silicon, which exhibits large changes under intense illumination. We explain the experimental data with a time domain, hydrodynamic-Maxwell approach broadly applicable to most optical materials. Our approach accounts simultaneously for surface and magnetic nonlinearities that generate *even optical harmonics*, as well as linear and nonlinear material dispersions beyond the third order to account for *odd optical harmonics*, plasma formation, and a phase locking mechanism that makes the generation of high harmonics possible deep into the UV range, where semiconductors like silicon start operating in a metallic regime.

## 1 Introduction

The last decade has witnessed a renewed quest for efficient nonlinear frequency conversion implementing micrometric and nanometric size materials and devices that have highlighted the importance of nanostructures and metasurfaces. This pursuit has been marked by a dramatic increase in theoretical and experimental work [1], [2], [3], [4], [5], [6], [7], [8], [9], [10], [11], [12] that has led to a natural reassessment of the optical properties of semiconductors and metals in optical regimes that were previously deemed impractical or inaccessible [13], [14], [15], [16], [17], [18], [19], [20], [21]. This kind of research has generally been limited to second and third harmonic generation (SHG and THG, respectively) primarily due the applicability of these processes to surface sensing, label-free bio-imaging, and quantum optics [22], [23], [24], [25]. However, nonlinear high harmonic generation (HHG) encompassing visible light, ultraviolet (UV), and even shorter wavelengths, is also of fundamental interest for other key applications such as ultra-fast physics, and X-UV photolithography [10], [26]. Most work on HHG has been carried out in transparent conductive oxides (TCOs) [27], [28], [29], [30] and all dielectric structures [5], [6] for the purpose of avoiding intrinsic losses associated with semiconductors and metals [31]. This has led researchers to not only set metals aside but also to focus almost exclusively on the transparency spectral range of dielectric and semiconductor materials.

Generally, the study of nonlinear optics at the nanoscale requires modifications of the material equations of motion to account for effects that manifest themselves only at the atomic level, given that the average atomic diameter is of order 3 Å–5 Å. Examples are linear and nonlinear nonlocal effects due to pressure and viscosity of the free electron fluid and spill-out effects [32], [33], [34], [35], [36], as well as tunnelling [37], [38], screening and surface and magnetic phenomena [39], [40], [41]. Even in transparent materials, most of the work has focused on bulk nonlinearities, while neglecting crucial surface and magnetic phenomena that in centrosymmetric materials have been traditionally associated with the generation of even-order harmonics [39]. Most semiconductors have broad absorption resonances deep in the UV range, perhaps suggesting that absorption may not be circumvented, with negative dielectric constants and epsilon near zero (ENZ) crossing points in the 100 nm range. However, it has been demonstrated both theoretically and experimentally that SHG and THG can occur despite absorption in the visible and UV ranges and can be transmitted through half millimeter thick wafers of silicon (Si), gallium arsenide (GaAs), and gallium phosphide (GaP) with the pump tuned in the transparency regime, via a phenomenon referred to as *phase-locking* [13], [14], [15], [16]. Although for nanometer-thick materials the conversion process is inefficient due to the absence and loss of meaning of phase matching, efficiency can be improved dramatically if a resonant mechanism comes into play as demonstrated for semiconductor cavities [17], and at least in theory in GaAs-filled metal gratings [18] and silicon nanowire arrays [19]. This phenomenon plays a critical role in nonlinear optical phenomena for harmonic generation in all semiconductors in the opacity range, and so we now briefly delve into the fundamental reasons that make this possible.

As originally pointed out by Bloembergen and Pershan [42] for SHG in transparent media, Maxwell’s equations in a nonlinear material have two solutions: a homogeneous component that propagates with a wave vector $k_{2\omega }^{\left(\mathrm{hom}\right)}=n\left(2\omega \right)2\omega /c$ , and an inhomogeneous component having wave vector $k_{2\omega }^{\left(inh\right)}=2k_{\omega }=2n\left(\omega \right)\omega /c$ . These distinct solutions can be generalized to the *j*th harmonic. In the plane wave regime and oblique incidence, these waves refract at different angles, interfere, and give rise to Maker fringes [43], with the inhomogeneous portion propagating at the same refraction angle and velocity as the pump beam, a phenomenon more recently clearly observed in bulk lithium niobate [44]. However, if the interaction occurs in such a way that the pump is tuned in the transparency range [ $n\left(\omega \right)$ is real], and the harmonic field is tuned in the opaque region [ $n\left(2\omega \right)$ is complex], then the homogenous component is absorbed, leaving behind the inhomogeneous component, setting up an anomalous situation, i.e., a harmonic signal that propagates with the same dispersion and group velocity of the pump, a phenomenon that has been referred to as phase locking, given the phase relationships between pump and the relevant inhomogeneous harmonic solutions [13], [14], [15], [16], [17], [18], [19], [20], [44].

Thanks to phase locking, a harmonic field tuned in the opaque regime can resonate along with the pump, as if absorption were not present for that component, provided the pump is tuned in the transparency range. This is the fundamental mechanism that explains the apparent suppression of absorption observed, for example in references [8], [9], and most recently highlighted and reported in the opacity range of a chalcogenide glass grating at 354 nm [45], well inside the band gap, and in silicon etalons [46], with THG conversion efficiencies of order 10^−7^ at 266 nm, with a pump intensity of 1.5 GW/cm^2^. The remarkable facts here are that one is exploiting the intrinsic properties of silicon without local field amplification [see Supplementary Information], and that silicon is metallic [Re(*ε*) < 0] between 100 nm and 300 nm. This efficiency should be contrasted with purely experimental observations of THG from three-dimensional (3-D), resonant silicon metasurfaces characterized by guided mode resonances and bound states in the continuum [47], [48]; where similar peak power densities yield THG conversion efficiencies of order 10^−4^. In the present case, using the same theory illustrated in references [13], [19], [46], we analyze experimental results that yield high harmonic generation in silicon using a time-domain, hydrodynamic-Maxwell pulse propagation method that considers linear and nonlinear bulk material dispersions, nonlinearities triggered by the mere traversal of surfaces, and by the intrinsically nonlinear magnetic portion of the Lorentz force. As we will see below, the confluence of the above-listed physical phenomena enables an accurate theoretical description of the relevant qualitative and quantitative aspects of nonlinear frequency conversion in the perturbative regime. We note that it is possible to extend the method to the non-perturbative regime, which we define as a harmonic oscillator far from its ground state, requiring adopting additional polarization and inversion components generated by a system of Bloch equations that describe a two- or multi-level atom, as shown in references [49], [50], where it was applied to stimulated Raman scattering near plasmonic structures.

The paper is organized as follows: in Section 2 we will first introduce equations of motion (Eqs. (1) and (2)) that describe the two absorption (Lorentzian) resonances that characterize silicon in the UV range. In Eq. (3) we then introduce the dynamics of conduction electrons, whose density is proportional to the incident field intensity, and which are mostly responsible for even harmonic generation at large peak power densities via surface (Coulomb, convective terms, and Lorentz magnetic force) and volume (Lorentz magnetic force) nonlinear sources. Unlike other approaches where high harmonic generation is also tackled theoretically [51], [52], our approach includes surface and magnetic nonlinearities, also described in some detail for both free and bound charges, which are essential for the generation of even harmonic fields in centrosymmetric materials. Finally in Section 2 we also describe our experimental measurements, compare with the results of our simulations, and offer a closing summary of our present effort.

## 2 Theory and results

**Figure 1:**
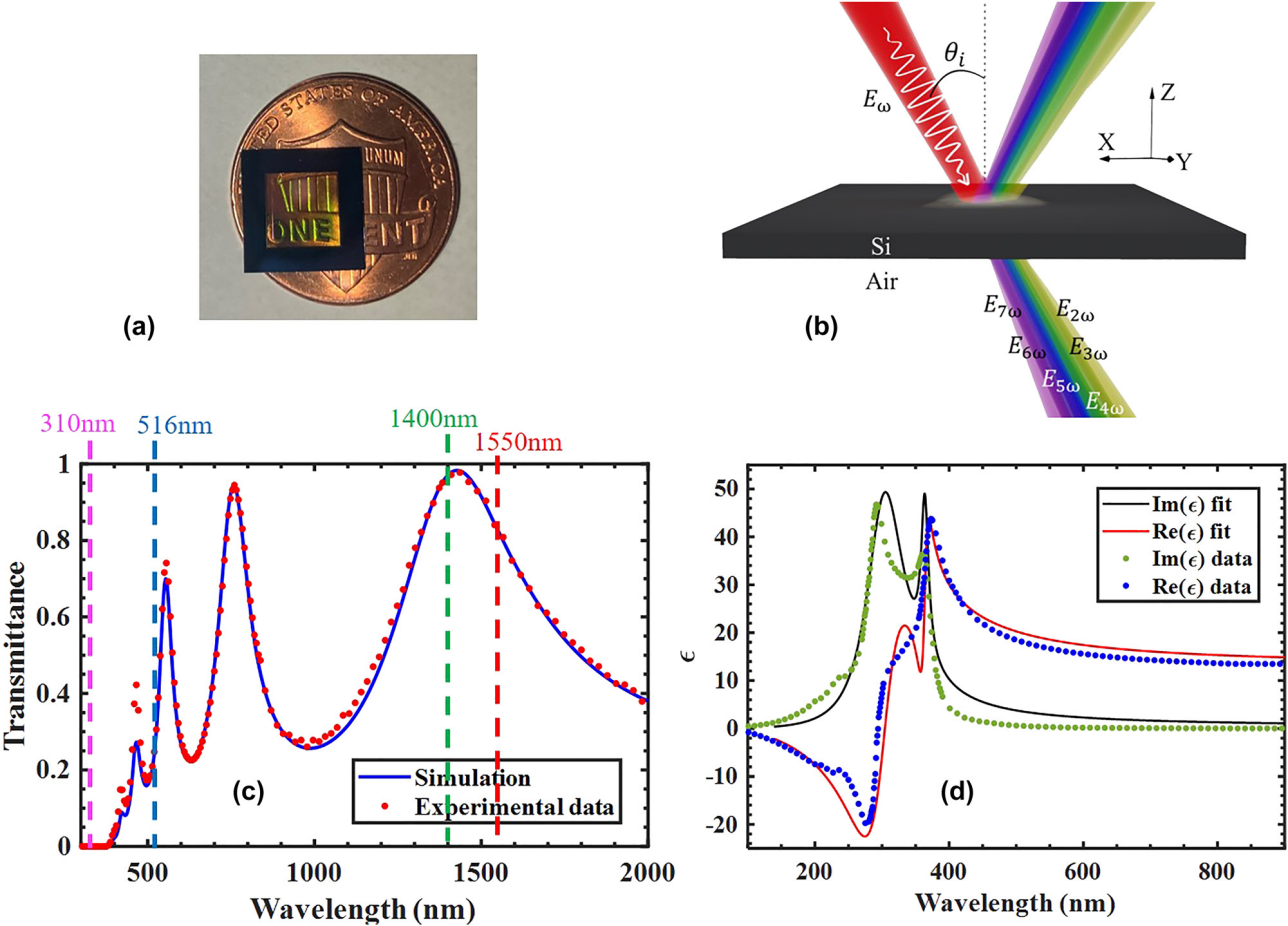
Schematic and linear properties of the silicon membrane. (a) Picture of a 200 nm-thick silicon film. The surface area of the suspended portion of the film (the area with greenish hue) is approximately 5 mm × 5 mm. (b) A pulse is incident from the top. We monitor only transmitted fields. (c) Linear transmittance measured at normal incidence (red markers) and fitted (blue curve) using Palik’s data in reference [31]. Some wavelengths of interest are marked in the figure. (d) Dielectric function of silicon as reported in reference [31] (markers) and as fitted by two Lorentzian functions (solid curves.)

The suspended sub-wavelength silicon films used in our experiments are pictured and schematically depicted in Figure 1(a) and (b). The resonance near 530 nm gives the sample a characteristic green hue. They were purchased from Norcada (Alberta, Canada) and consist of ∼200 nm-thick <100> silicon film with doping level of order 10^16^/cm^3^, etched out of a silicon carrier wafer, and held by a frame of proprietary composition. This density corresponds to a plasma frequency in the THz range and does not affect the dynamics at low intensities. We will return to the subject later under peak intensity conditions. In Figure 1(c) we report both measured and fitted transmittance at normal incidence. The etalon displays a series of Fabry–Perot resonances whose amplitude expectedly decreases rapidly with decreasing wavelength. Our measurements are well represented by Palik’s data [31], which we show in Figure 1(d) along with a fit obtained using two detuned Lorentzian functions that we will use in our simulations. The sample is completely opaque below 400 nm, erroneously suggesting that we should not expect any meaningful nonlinear response in this range. The dielectric function displays rapid variations below 500 nm. Undoped silicon is metallic below 300 nm (shaded region), and its dispersion is marked by two adjacent resonances in the UV range [13], [31], [46], which according to Miller’s rule [53], [54] give rise to resonant nonlinearities in the opaque range. The system may then be described by two polarization components having two closely spaced resonance frequencies and separate linear and nonlinear spring constants, as follows:

(1) $$\begin{array}{cc} & {\ddot{P}}_{bj}+{\tilde{\gamma }}_{bj}{\ddot{P}}_{bj}+{\tilde{\omega }}_{0,bj}^{2}P_{bj}-{\tilde{\beta }}_{bj}\left(P_{bj}\cdot P_{bj}\right)P_{bj}\\ & +{\tilde{\delta }}_{bj}{\left(P_{bj}\cdot P_{bj}\right)}^{2}P_{bj}-{\tilde{\theta }}_{bj}{\left(P_{bj}\cdot P_{bj}\right)}^{3}P_{bj}\\ & \;=\pi {\tilde{\omega }}_{pj}^{2}E+\frac{e\lambda_{r}}{m_{bj}^{*}c^{2}}\left(P_{bj}\cdot \nabla \right)E+\frac{e\lambda_{r}}{m_{bj}^{*}c^{2}}{\dot{P}}_{bj}\times H \end{array}$$

Here, $m_{bj}^{*}$ is the effective bound electron mass for the *j*th polarization component; ${\tilde{\omega }}_{0,bj}=\omega_{0,bj}\lambda_{r}/c$ , ${\tilde{\omega }}_{pj}=\omega_{pj}\lambda_{r}/c$ , ${\tilde{\gamma }}_{bj}=\gamma_{bj}\lambda_{r}/c$ are the scaled resonance and plasma frequencies, respectively, and damping coefficient; *λ*_*r*_ = 1 μm is a reference wavelength that scales *ξ* = *z*/*λ*_*r*_, *ζ* = *x*/*λ*_*r*_, *ς* = *y*/*λ*_*r*_ where *x* and *y* are the transverse coordinates, and *z* is the longitudinal coordinate; *τ* = *ct*/*λ*_*r*_ is the scaled time, and *c* is the speed of light in vacuum. Spatial and temporal derivatives on the fields are performed with respect to these new scaled spatial and temporal coordinates. The left-hand side of Eq. (1) contains internal linear and nonlinear restoring forces, while on the right-hand side we have forces resulting from externally applied fields.

Since we consider up to 7th harmonic generation, the bulk nonlinear response from bound electrons must reflect at least a 7th order nonlinearity, and may be written as:

(2) $$\begin{array}{ll} P_{NL,j} & =-{\tilde{\beta }}_{bj}\left(P_{bj}\cdot P_{bj}\right)P_{bj}+{\tilde{\delta }}_{bj}{\left(P_{bj}\cdot P_{bj}\right)}^{2}P_{bj}\\ & \;-{\tilde{\theta }}_{bj}{\left(P_{bj}\cdot P_{bj}\right)}^{3}P_{bj} \end{array}$$

Material response is assumed to be isotropic but could easily be modified to account for anisotropies [55] by isolating the spatial coordinates and by assigning the coefficients different values in different directions. In reference [55], which to our knowledge is the first report of reflected THG from silicon in the UV range, the authors investigated the question of THG using a nanosecond pump tuned to 1,064 nm and found an effective anisotropy in the third order nonlinear response. In the low intensity regime (which we arbitrarily set at 100 GW/cm^2^ or less) integration of Eq. (1) together with Maxwell’s equations account for linear and nonlinear dispersions, and allow us to describe the process by considering the consequences of terms like $\frac{e\lambda_{r}}{m_{0,bj}^{*}c^{2}}\left(P_{bj}\cdot \nabla \right)E$ , which represents surface nonlinearities, and the magnetic Lorentz contribution $\frac{e\lambda_{r}}{m_{0,bj}^{*}c^{2}}{\dot{P}}_{bj}\times H$ , which contains both surface and volume nonlinear bound currents. Both terms are also expanded up to their 7th harmonic contributions (not shown here), and partially account for even harmonic generation in centrosymmetric materials that act as insulators.

We now outline a novel approach to solving Eq. (1). The parameters ${\tilde{\beta }}_{bj}\approx \omega_{0,bj}^{2}\lambda_{r}^{2}/\left(L^{2}n_{0,bj}^{2}e^{2}c^{2}\right)$ , ${\tilde{\delta }}_{bj}={\tilde{\beta }}_{bj}/\left(L^{2}n_{0,bj}^{2}e^{2}\right)$ , and ${\tilde{\theta }}_{bj}={\tilde{\beta }}_{bj}/\left(L^{4}n_{0,bj}^{4}e^{4}\right)$ are higher order scaled coefficients in a perturbative expansion that are derived directly from a nonlinear classical oscillator model, and are usually assumed to be constant, *n*_0,*bj*_ is the bound electron density, and *L* is usually interpreted as the lattice constant. From the point of view of a classical spring and classical macroscopic electrodynamics, the lattice constant *L* represents the spring’s maximum allowed extension, which is generally taken to be constant across the entire spectral range. From an atomic point of view, *L* may be interpreted as either an atomic/orbital diameter or inter-particle distance, which for solids can vary from a fraction of 1 Å to several Ås, a disparity that will be reflected in the particle density, and that will substantially affect the magnitudes of $\tilde{\beta }$ , $\tilde{\delta }$ , and $\tilde{\theta }$ . *However, since harmonic generation occurs over a wide wavelength range, all the parameters outlined above are unlikely to remain constant, including electronic effective masses, due to the different energy levels that are found deeper and deeper inside the atom, with complex and primarily unknown orbital radii, population density, and band curvatures*. Therefore, orbitals and energy levels that are excited at the pump wavelength are different compared to states excited at each harmonic wavelength, will have different diameters and effective particle densities depending on the number of electrons in each orbital, so that at least the parameters *L* and *n*_*0*_ at the pump wavelength may be different at each of the harmonics. From a classical, macroscopic point of view, retrieval of $\tilde{\beta }$ , $\tilde{\delta }$ and $\tilde{\theta }$ should be done in the same manner that the dielectric constant is retrieved, i.e. via ellipsometric techniques for a given thickness and wavelength. Then, it is reasonable to expect that *ɛ*, $\tilde{\beta }$ , $\tilde{\delta }$ , and $\tilde{\theta }$ will display dispersive behavior not only as a function of wavelength, but also as a function of geometrical dispersion (resonances) [56]. Therefore, while it is generally acceptable to assume that $\tilde{\beta }$ is constant under most circumstances [46], [53], [54], we may well have $\tilde{\beta }={\tilde{\beta }}_{0}f_{\tilde{\beta }}\left(\lambda \right)$ . Here, ${\tilde{\beta }}_{0}=\omega_{0,bj}^{2}\lambda_{r}^{2}/\left(L_{0}^{2}n_{0,bj}^{2}e^{2}c^{2}\right)$ is defined using the known lattice constant and particle density for the material, and $f_{\tilde{\beta }}\left(\lambda \right)$ is a parameter that generalizes the third order nonlinear coefficient in Eq. (1) that reflects the changing nature of the material across the spectrum. By the same token, we will assume that $\tilde{\delta }={\tilde{\delta }}_{0}f_{\tilde{\delta }}\left(\lambda \right)$ and $\tilde{\theta }={\tilde{\theta }}_{0}f_{\tilde{\theta }}\left(\lambda \right)$ , with ${\tilde{\delta }}_{0}={\tilde{\beta }}_{0}/\left(L_{0}^{2}n_{0,bj}^{2}e^{2}\right)$ and ${\tilde{\theta }}_{0}={\tilde{\beta }}_{0}/\left(L_{0}^{4}n_{0,bj}^{4}e^{4}\right)$ . Finally, given the proximity of the material resonances in Figure 1(d), for simplicity we will assume that the coefficients ${\tilde{\beta }}_{bj}$ , ${\tilde{\delta }}_{bj}$ , and ${\tilde{\theta }}_{bj}$ have similar amplitudes at both resonance wavelengths.

As we will see below, observation of the 7th harmonic at 210 nm using our detection system will require peak power densities of order 3 TW/cm^2^. At these power densities and 10 Hz repetition rate our incident beam delivers an energy density of approximately 270 mJ/cm^2^. Nevertheless, we expect single-pulse effects given the temporal displacement between consecutive pulses, each containing approximately 27 mJ/cm^2^. The threshold for plasma formation in 3 μm-thick Si layers has been reported to be of order 150 mJ/cm^2^ when it is delivered within ∼200 fs using optimized pulses and yielding remarkably high free electron densities of order 10^22^–10^23^/cm^3^ [57]. In our case, the sample survives undamaged if we keep peak power densities below 4 TW/cm^2^. Our calculations suggest free carrier densities of order 10^19^–10^20^/cm^3^. Plasma formation requires an additional, transient polarization component ***P***_*f*_ to include a description of the dynamics of free carriers. This component triggers additional effects like pump shielding and an intensity dependent plasma frequency (increasing carrier density) that tends to blueshift as a function of incident peak power density. In turn this lowers the effective dielectric constant dynamically, affecting the structure and position of the geometrical resonances, including modifying the coefficients $\tilde{\beta }$ , $\tilde{\delta }$ , and $\tilde{\theta }$ , which continue to be dispersive and have to be modified accordingly. Put another way, the increased free charge density comes at the expense of a decreasing bound charge density, causing the coefficients $\tilde{\beta }$ , $\tilde{\delta }$ , and $\tilde{\theta }$ to change dynamically as a redistribution of charge occurs. Given these factors, the essential equation of motion that supplements Eq. (1) and describes the dynamics of free carriers may be written as follows [13], [40], [41], [46]:

(3) $$\begin{array}{ll} {\ddot{P}}_{f}+{\tilde{\gamma }}_{f}{\dot{P}}_{f} & =\left(\pi {\tilde{\omega }}_{p,f}^{2}+\kappa_{f}E\cdot E\right)E+\frac{e\lambda_{r}}{m_{f}^{*}c^{2}}\left(\nabla \cdot P_{f}\right)E\\ & \;+\frac{3}{5}\frac{E_{F}}{m_{f}^{*}c^{2}}\left[\nabla \left(\nabla \cdot P_{f}\right)+\frac{1}{2}\nabla^{2}P_{f}\right]\\ & \;+\frac{e\lambda_{r}}{m_{f}^{*}c^{2}}{\dot{P}}_{f}\times H-\frac{1}{n_{0f}e\lambda_{r}}\\ & \;\times \left[\left(\nabla \cdot {\dot{P}}_{f}\right){\dot{P}}_{f}+\left({\dot{P}}_{f}\cdot \nabla \right){\dot{P}}_{f}\right]. \end{array}$$

Therefore, we account for surface and magnetic phenomena in both free and bound charges. Plasma frequency ${\tilde{\omega }}_{p,f}$ and damping coefficients ${\tilde{\gamma }}_{f}$ are scaled similarly to their bound electron counterparts. The presence of the coefficient *κ*_*f*__,_ which may depend on fluence and may also be dispersive, allows the scaled plasma frequency ${\tilde{\omega }}_{p,f}$ to change as a function of intensity (blueshift) and time, with contributions to *all* the harmonics, as revealed by an expansion of the fields up to 7th harmonic. The term proportional to the Fermi energy, $E_{F}=\frac{\hbar^{2}}{2m_{f}^{*}}{\left(3\pi^{2}n_{0f}\right)}^{2/3}$ , contains the effects of linear pressure and viscosity, $m_{f}^{*}$ and *n*_0*f*_ are the free electron mass and background density, respectively. The terms in Eq. (3) thus reflect a continuity equation that yields contributions to an *effective plasma frequency* that depends on the local charge density ∇ ⋅ ***P***_*f*_ and a third order nonlinearity as follows [58]

(4) $$n\left(P,E\right)=\left(n_{0f}-\frac{1}{e}\nabla \cdot P_{f}+\kappa_{f}E\cdot E+\cdots \right).$$

Nonlocal terms $\left(\nabla \left(\nabla \cdot P_{f}\right)+\frac{1}{2}\nabla^{2}P_{f}\right)$ then make the dielectric function a highly dynamic variable.

**Figure 2:**
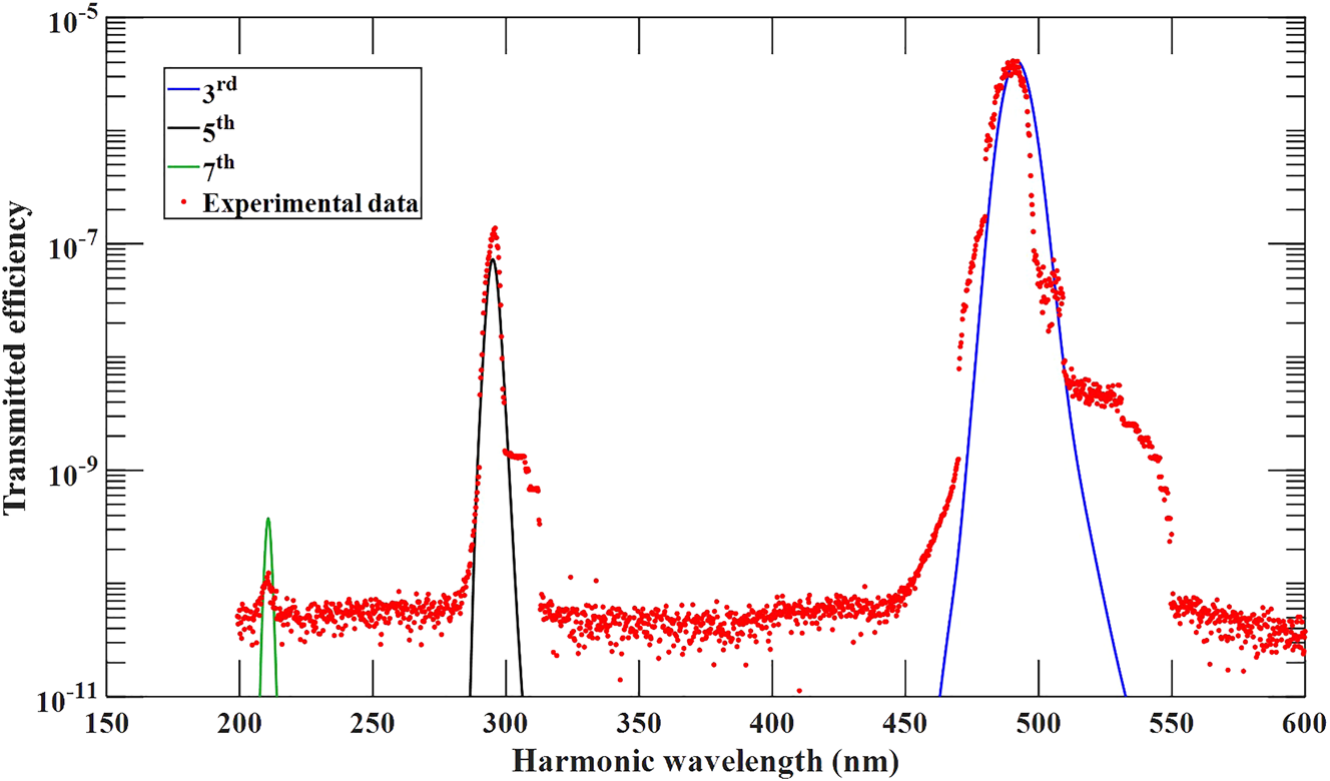
Measured and simulated spectra of transmitted 3rd, 5th, and 7th harmonic conversion efficiencies for a single pulse 85 fs in duration, having carrier wavelength tuned to 1,475 nm, peak power density of 3 TW/cm^2^, and normally incident on the silicon film. Relevant parameters: $\tilde{\beta }\left(\omega \right)=5\times 10^{-10}$ , $\tilde{\beta }\left(3\omega \right)=2\times 10^{-10}$ , $\tilde{\beta }\left(5\omega \right)=5\times 10^{-11}$ , $\tilde{\beta }\left(7\omega \right)=5\times 10^{-11}$ , $\tilde{\delta }\left(\omega \right)=1.25\times 10^{-19}$ , $\tilde{\delta }\left(3\omega \right)=5\times 10^{-20}$ , $\tilde{\delta }\left(5\omega \right)=1.25\times 10^{-20}$ , $\tilde{\delta }\left(7\omega \right)=1.25\times 10^{-20}$ , $\tilde{\vartheta }\left(\omega \right)=6.25\times 10^{-29}$ , $\tilde{\vartheta }\left(3\omega \right)=2.5\times 10^{-29}$ , $\tilde{\vartheta }\left(5\omega \right)=6.25\times 10^{-30}$ , $\tilde{\vartheta }\left(7\omega \right)=6.25\times 10^{-30}$ , $k_{f}\left(\omega \right)=1.6\times 10^{-9}$ , $k_{f}\left(3\omega \right)=6.4\times 10^{-10}$ , $k_{f}\left(5\omega \right)=1.6\times 10^{-10}$ , $k_{f}\left(7\omega \right)=1.6\times 10^{-10}$ .

In Figure 2 we show the measured and simulated spectra generated by a ∼3 TW/cm^2^, 85 fs pump pulse tuned to 1,475 nm incident normal to the surface to temporarily suppress even harmonics. The measured conversion efficiencies are 4.2 × 10^−5^, 6.2 × 10^−7^, and 4.57 × 10^−9^ for 3rd, 5th, and 7th harmonic, with peak power densities of order 126 MW/cm^2^, 1.8 MW/cm^2^ and 14 kW/cm^2^, respectively, despite being tuned in the absorption and metallic ranges. Agreement between the measured and simulated data is remarkable. It is worth mentioning that the experimentally recorded side lobes contain less than 0.1 % of their respective harmonic energy and can be attributed to small variations in temporal and spatial profiles of the experimental source when compared to the perfectly formed Gaussian pulses used in simulations. Phase-locking is the fundamental mechanism that makes this kind of interaction possible. Our simulations suggests that results like those depicted in Figure 2 can be obtained by tuning the pump field at each of the resonances shown in Figure 1(c), pushing the highest harmonic we can presently simulate below 100 nm. See Supplementary Information for a scan of 7th harmonic generation efficiency for a range of wavelength that includes the resonances shown in Figure 1(c).

**Figure 3:**
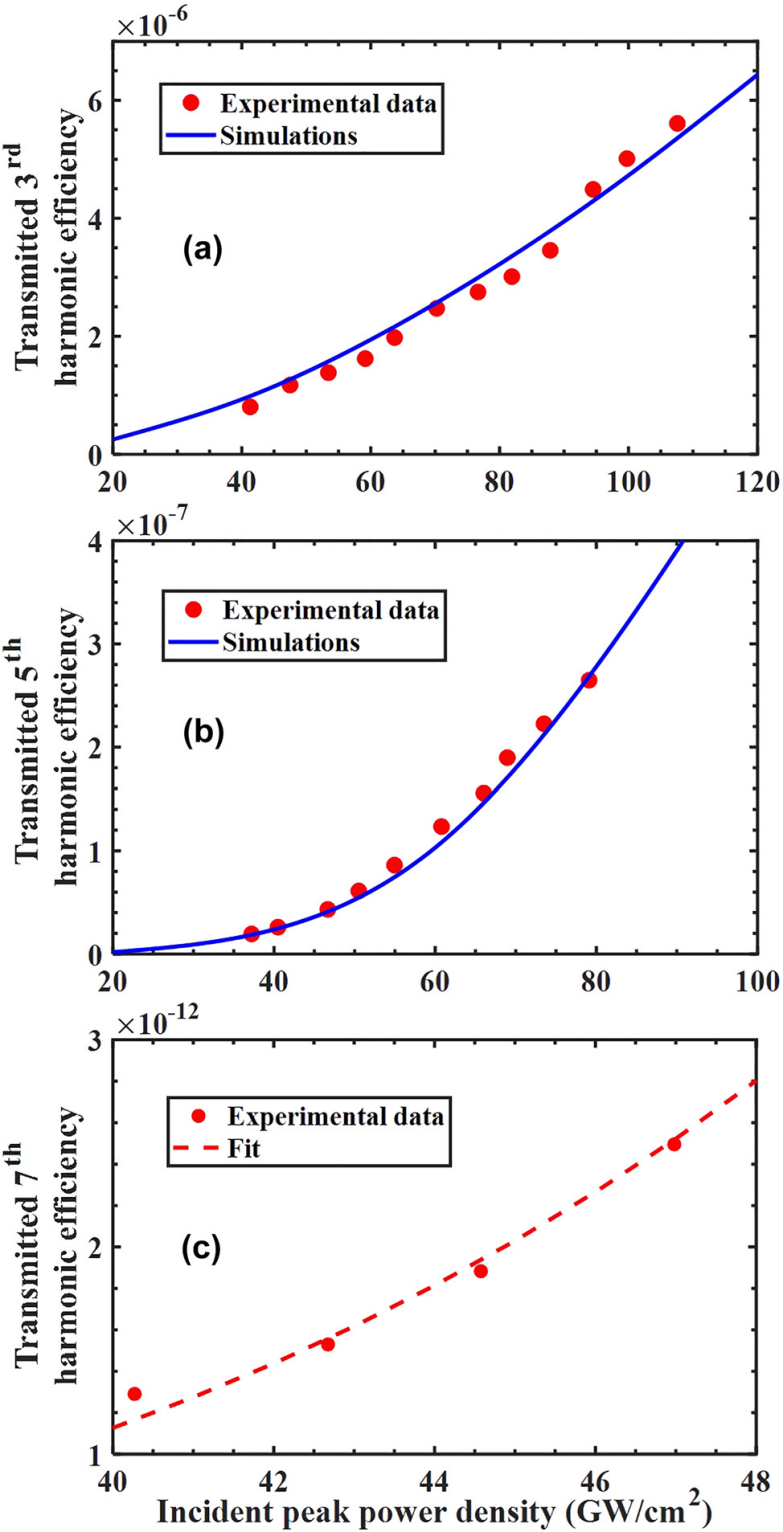
Transmitted third (a) and fifth (b) harmonic generation efficiency versus incident peak power density. An intensity scan is performed with a ∼100 fs pulses tuned to 1,550 nm with repetition rate 2 Hz. The 3rd (516 nm) and 5th (310 nm) harmonic data are fitted almost perfectly by the simulations. Relevant parameters: $\tilde{\beta }\left(3\omega \right)={\tilde{\beta }}_{0}=8.5\times 10^{-9}$ , $\tilde{\delta }\left(3\omega \right)={\tilde{\delta }}_{0}=4.25\times 10^{-18}$ , $\tilde{\vartheta }\left(3\omega \right)={\tilde{\vartheta }}_{0}=2.125\times 10^{-27}$ , $\tilde{\beta }\left(5\omega \right)={\tilde{\beta }}_{0}$ , $\tilde{\delta }\left(5\omega \right)=9{\tilde{\delta }}_{0}$ , $\tilde{\vartheta }\left(5\omega \right)=5{\tilde{\vartheta }}_{0}$ , $\tilde{\beta }\left(7\omega \right)=4{\tilde{\beta }}_{0}$ , $\tilde{\delta }\left(7\omega \right)={\tilde{\delta }}_{0}$ , $\tilde{\vartheta }\left(7\omega \right)={\tilde{\vartheta }}_{0}$ . (c) Measured 7th harmonic conversion efficiency versus peak power density for the setup up in Figure S1(a). The fit follows the 5th power of the peak power density. The field is tuned off resonance to 2,150 nm, is incident at 30°, with 20 Hz repetition rate.

We then pump the sample at 1,550 nm at normal incidence and perform an intensity scan to detect *third* and *fifth* harmonic signals at 516 nm and 310 nm, which is our detection limit for the low-intensity setup. In Figure 3(a) and (b) we show the result of both scans, along with the result of our simulations. Conversion efficiency is defined by dividing the total energy of a given harmonic by the total incident pump energy. Once the dielectric constant is established and fitted using the linearized version of Eq. (1) [13], $\tilde{\beta }$ and $\tilde{\delta }$ are the only remaining parameters to be determined. The reconstruction of nonlinear dispersion when $\tilde{\delta }$ is not zero is complicated because the $\tilde{\delta }$ terms tend to quench the $\tilde{\beta }$ contributions at both the pump and its harmonic wavelengths, given their opposite signs. This effect is discussed in reference [59]. The detection system in Figure S1(a) can be used to detect signals down to approximately 310 nm. Therefore, a 7th harmonic was also observed when the pump pulse was tuned to a carrier wavelength of 2,150 nm. We show the measured 7th harmonic efficiency versus incident peak power density in Figure 3(c). We note that the transmission function in Figure 1(c) expresses the behavior of a *pump field* tuned to that wavelength and says nothing about the behavior of a harmonic signal generated and tuned to the same wavelength. This conclusion is fully supported by the fact that the generated 5th harmonic signal is transmitted by the etalon, notwithstanding the fact that the linear transmission function suggests that wavelength should be fully suppressed. We emphasize that transmission of the 210 nm and 310 nm signals occurs because they are the inhomogeneous components of the generated harmonics, which propagate under phase locking conditions, i.e., with the dispersive properties of the pump field tuned in the transparency range of the material.

**Figure 4:**
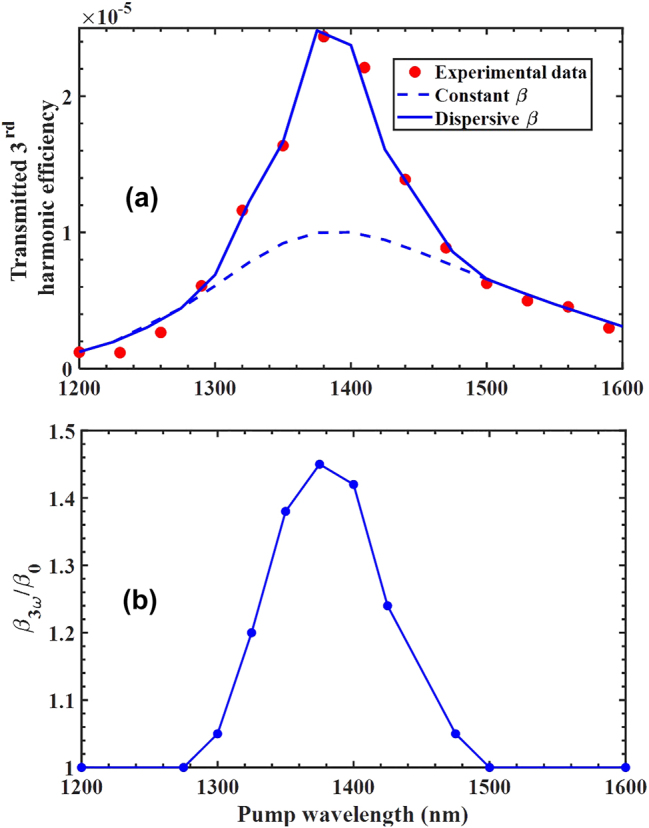
Transmitted THG conversion efficiency for (a) constant *β* (dashed blue curve) and with a *β*_*3ω*_ that varies as a function of wavelength (solid blue curve) as in (b). The red markers represent the measured data. (b) Actual functional dependence of the coefficient *β*_*3ω*_ used at the third harmonic wavelength. Relevant parameters: $\tilde{\beta }\left(3\omega \right)={\tilde{\beta }}_{0}=8.5\times 10^{-9};\tilde{\delta }\left(3\omega \right)={\tilde{\delta }}_{0}=4.25\times 10^{-18};\tilde{\vartheta }\left(3\omega \right)={\tilde{\vartheta }}_{0}=2.125\times 10^{-27}$ , $\tilde{\beta }\left(5\omega \right)={\tilde{\beta }}_{0};\tilde{\delta }\left(5\omega \right)=9{\tilde{\delta }}_{0};\tilde{\vartheta }\left(5\omega \right)=5{\tilde{\vartheta }}_{0}$ , $\tilde{\beta }\left(7\omega \right)=4{\tilde{\beta }}_{0};\tilde{\delta }\left(7\omega \right)={\tilde{\delta }}_{0};\tilde{\vartheta }\left(7\omega \right)={\tilde{\vartheta }}_{0}$ .

We now focus on the geometrical resonance centered near 1,400 nm and scan the carrier wavelength of the pump field across it. The result is plotted in Figure 4, where we show measurements and simulations of the THG conversion efficiency as a function of wavelength, as well as the dispersive character of $\tilde{\beta }$ across the resonance – Figure 4(b). Figure 4(a) contains two simulated curves, one obtained with a constant $\tilde{\beta }$ at all wavelengths, and one calculated using the dispersive coefficient ${\tilde{\beta }}_{3\omega }\left(\lambda \right)$ shown in Figure 4(b). While a constant $\tilde{\beta }$ yields good qualitative agreement across the resonance, it is only when using our full dispersive model that qualitative and quantitative agreement can be achieved. This is clearly shown in Figure 4(a) by a direct comparison of experiment and the two models with constant and dispersive ${\tilde{\beta }}_{3\omega }$ , respectively. At resonance, the dispersive coefficient ${\tilde{\beta }}_{3\omega }$ is nearly 1.5 times larger compared to its unitary value off resonance, shown in Figure 4(b). The experimental results clearly point towards a dispersive variation of the model and thus reveal that at least from a classical, macroscopic electrodynamics point of view, theory can be easily reconciled with measurements if we assume that $\tilde{\beta }$ is dispersive across the resonance.

**Figure 5:**
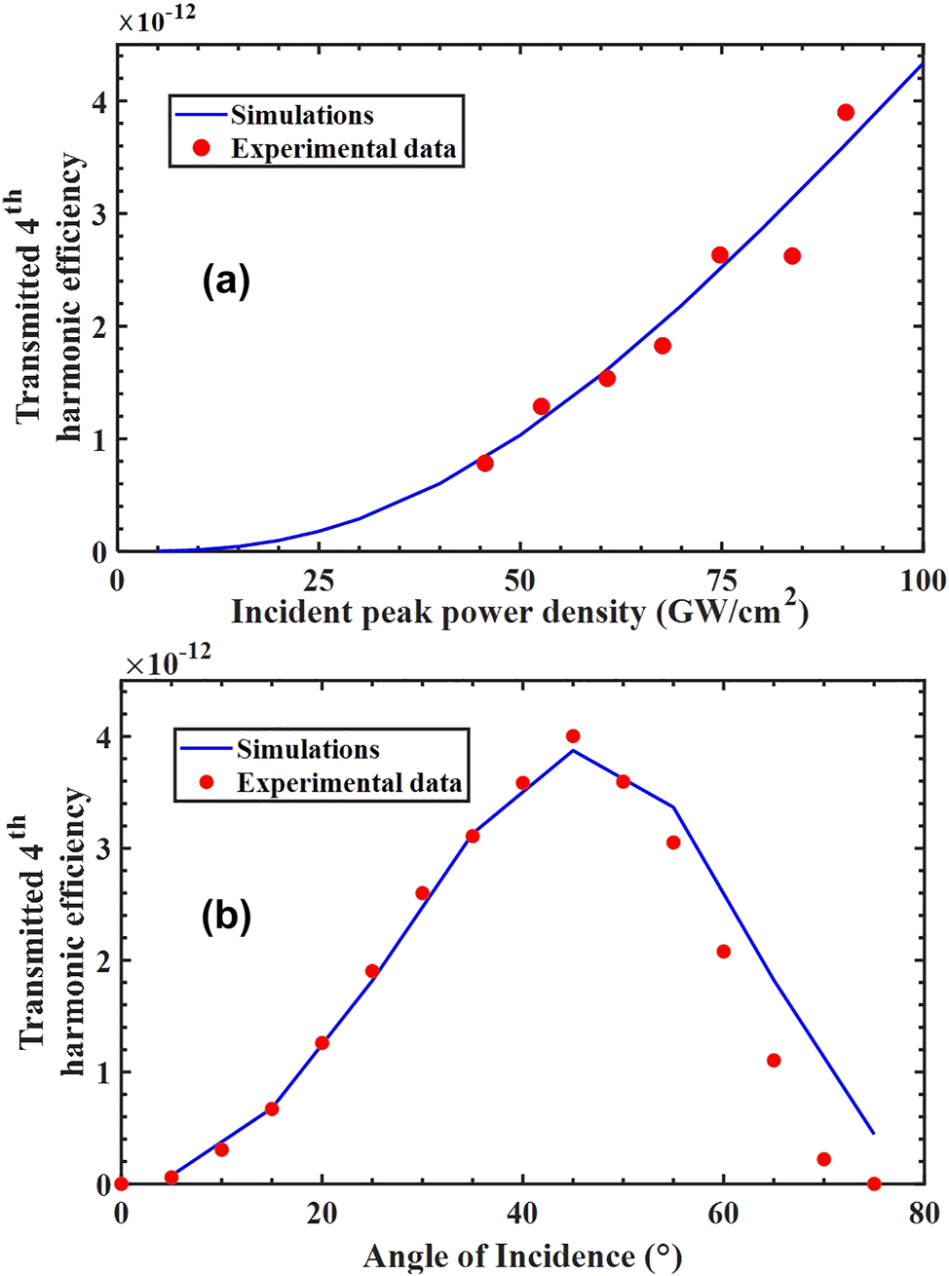
Transmitted (a) 4th harmonic generation conversion efficiency versus incident peak power density with angle of incidence fixed at 30°, carrier wavelength of 1,550 nm and repetition rate of 20 Hz. The red solid markers denote the data, while the simulations are reported as a solid blue curve. (b) Measured and simulated angular dependence of the 4th harmonic signal as a function of incident angle for fixed incident peak power density of 75 GW/cm^2^. Data are shown with red markers and simulations are shown as blue solid curves. Relevant parameters: $m_{b}^{*}\left(2\omega \right)=0.24m_{e};m_{b}^{*}\left(4\omega \right)=0.002m_{e};\tilde{\beta }=1.5\times 10^{-9};\tilde{\delta }=2.25\times 10^{-18};\tilde{\vartheta }=2.25\times 10^{-29}$ .

In Figure 5(a) we show 4th harmonic generation conversion efficiency as a function of incident peak power density for fixed angle. In Figure 5(b) we fix the incident peak power density and perform an angular scan to reveal the angular dependence of the 4th harmonic. Once again, the agreement between experiment and theory is remarkable and, to the best of our knowledge, unprecedented. The only free parameters are the effective free electron masses at the different harmonic wavelengths, and the bulk coefficients in Eq. (2). SHG results for similar samples were previously reported [46], while the 6th harmonic was not detectable for the intensities we used in this setup (up to 100 GW/cm^2^).

**Figure 6:**
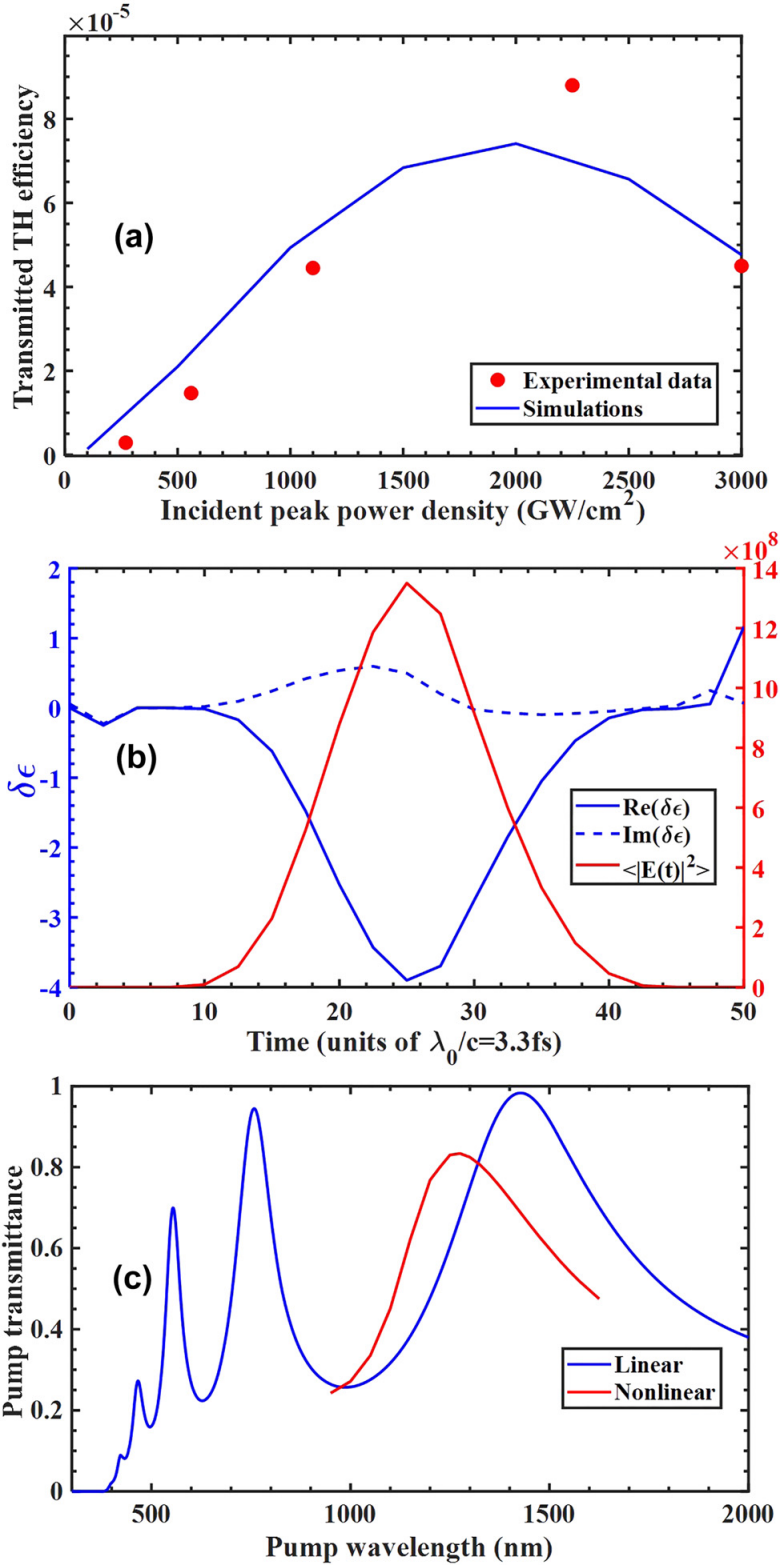
Measured and simulated (a) transmitted THG efficiency at high intensities. Pump is tuned at 1,475 nm with a pulse duration of 85 fs; (b) simulated extraction of the effective dielectric constant from the constitutive relations following the procedure developed in reference [13], for peak power densities of order 1 TW/cm^2^. (c) Linear (blue curve) and nonlinear (red curve) transmission functions showing the blueshift of the resonance for a 200 nm thick etalon. The relevant parameters are: $\tilde{\beta }\left(\omega \right)=5\times 10^{-10};\tilde{\beta }\left(3\omega \right)=2\times 10^{-10};\tilde{\beta }\left(5\omega \right)=5\times 10^{-11};\tilde{\beta }\left(7\omega \right)=5\times 10^{-11}$ , $\tilde{\delta }\left(\omega \right)=1.25\times 10^{-19};\tilde{\delta }\left(3\omega \right)=5\times 10^{-20};\tilde{\delta }\left(5\omega \right)=1.25\times 10^{-20};\tilde{\delta }\left(7\omega \right)=1.25\times 10^{-20}$ ; $\tilde{\vartheta }\left(\omega \right)=6.25\times 10^{-29};\tilde{\vartheta }\left(3\omega \right)=2.5\times 10^{-29};\tilde{\vartheta }\left(5\omega \right)=6.25\times 10^{-30};\tilde{\vartheta }\left(7\omega \right)=6.25\times 10^{-30}$ ; $k_{f}\left(\omega \right)=1.6\times 10^{-9};k_{f}\left(3\omega \right)=6.4\times 10^{-10};k_{f}\left(5\omega \right)=1.6\times 10^{-10};k_{f}\left(7\omega \right)=1.6\times 10^{-10}$ .

In Figure 6(a) we plot transmitted THG conversion efficiency versus incident peak power density for the 85 fs pulse tuned to 1,475 nm. Both measured data and simulation display a maximum near 2 TW/cm^2^, followed by a decrease in efficiency. We refer to Figure 6(b) and (c) to explain this behavior. In Figure 6(b) we simulate and plot the effective dielectric constant as a function of time as the pulse traverses the thin film, for a peak power density of approximately 1 TW/cm^2^. The simulation reveals a maximum change of order $Re\left(\delta \varepsilon \right)∼-4$ when the peak of the pulse reaches the center of the layer. The extraction of this parameter and the method is described in detail in reference [13] and requires knowledge of the constitutive relations. Suffice it to say here that in essence we perform linear and nonlinear numerical ellipsometry on the sample, by calculating the macroscopic dielectric constant as $\left\langle \varepsilon \left(t\right)\right\rangle =1+4\pi \left\langle \frac{P\left(t\right)}{E\left(t\right)}\right\rangle$ , where the brackets stand for spatial average inside the layer at each instant of time. This definition coincides with the result of an experimental ellipsometric measurement, as demonstrated in reference [13]. Then, the complex change of the dielectric constant may be calculated as the difference between the high and the low intensity dielectric functions as follows: $\left\langle \delta \varepsilon \right\rangle =\left\langle \varepsilon_{NL}\right\rangle -\left\langle \varepsilon_{L}\right\rangle =4\pi \left[\left\langle \frac{P_{NL}}{E_{NL}}\right\rangle -\left\langle \frac{P_{L}}{E_{L}}\right\rangle \right]$ , where the subscript *L* and *NL* stand for linear and nonlinear, respectively. A dynamic reduction of the dielectric constant from approximately 13 to 9 at the pump wavelength causes an intensity dependent blueshift of the resonance, shown in Figure 6(c), effectively detuning the pump out of resonance and reducing the conversion efficiency.

Figure 6(b) reveals an exceptionally powerful spatio-temporal dynamics, i.e., time varying capability that may rival and even surpass that of transparent conductive oxides by exploiting resonant nonlinearities and plasma formation. While conductive oxides may seem to hold the advantage in transparency, the transmission resonances and field localization that can be created in a Fabry–Perot cavity or in nanowire arrays [19] may also lead silicon and other semiconductors to serve as alternative building blocks of time varying metamaterials.

At this point, a note about parameter space is in order. The total nonlinear bulk polarization that is inserted into Maxwell’s equation is the sum of the combined solutions of Eqs. (1) and (3), while the instantaneous nonlinear potential may be thought of as a combination of free and bound polarizations as follows:

(5) $$\begin{array}{ll} P_{NL,j} & =\kappa_{f}\left(E\cdot E\right)E+{\tilde{\beta }}_{bj}\left(P_{bj}\cdot P_{bj}\right)P_{bj}\\ & \;-{\tilde{\delta }}_{bj}{\left(P_{bj}\cdot P_{bj}\right)}^{2}P_{bj}+{\tilde{\theta }}_{bj}{\left(P_{bj}\cdot P_{bj}\right)}^{3}P_{bj} \end{array}$$

When the fields and polarizations are expanded up to the 7th harmonic frequency, each term in Eq. (5) contributes to each of the harmonics, including even harmonics. For instance, some of the terms that oscillate at the fundamental wavelength are proportional to $\kappa_{f}{\left|E_{\omega }\right|}^{2}E_{\omega }$ , ${\tilde{\beta }}_{j}{\left|P_{\omega }\right|}^{2}P_{\omega }$ , ${\tilde{\delta }}_{bj}{\left|P_{\omega }\right|}^{4}P_{\omega }$ , and ${\tilde{\theta }}_{bj}{\left|P_{\omega }\right|}^{6}P_{\omega }$ . Similarly, just a few of the terms that oscillate at the second harmonic are $\kappa_{f}{\left|E_{\omega }\right|}^{2}E_{2\omega },\;{\tilde{\beta }}_{j}{\left|P_{\omega }\right|}^{2}P_{2\omega }$ , ${\tilde{\delta }}_{bj}P_{\omega }^{4}P_{2\omega }^{*}$ , and ${\tilde{\vartheta }}_{bj}\left|P_{\omega }\right|P_{\omega }^{4}P_{2\omega }^{*}$ . At the third harmonic wavelength some of the terms are proportional to $\kappa_{f}E_{\omega }^{3}$ , ${\tilde{\beta }}_{j}P_{\omega }^{3}$ , ${\tilde{\delta }}_{bj}{\left|P_{\omega }\right|}^{2}{P_{\omega }}^{3}$ , ${\tilde{\theta }}_{bj}{\left|P_{\omega }\right|}^{4}P_{\omega }^{3}$ . The leading cubic terms can balance each other, while 5th and 7th powers of the fields tend to either quench or reinforce terms of lower order, depending on the magnitude of the coefficients, as well as material dispersion, since the polarizations intrinsically contain the susceptibilities, which may be either positive or negative depending on tuning. Therefore, the set of parameters that we use in our simulations can be modified somewhat and still yield similar results.

## 3 Conclusions

In summary, we have reported odd and even nonlinear frequency conversion up to the 7th harmonic from a subwavelength silicon film. Several of the harmonics are generated in the opacity range, where the material displays metallic behavior with a negative dielectric constant. Those harmonics are transmitted thanks to a phase locking mechanism that allows the inhomogeneous component to effectively defeat nonlinear absorption as it resonates and propagates through thick layers, if the pump is tuned in the transparency range. Our measurements and simulations show that harmonic generation deep in the UV range is possible, can be efficient under resonant conditions [19], 46], and Supplementary Information], and the sample can withstand peak power densities well above 1 TW/cm^2^. It is remarkable that the reported conversion efficiencies are quite high deep in the UV range, where the material is metallic and absorptive. It is notable that the efficiencies observed for silicon membranes, although impressive and somehow unexpected, are still at least one order of magnitude lower (across all harmonics) than those reported in transparent conductive oxides films pumped near the ENZ crossover wavelength [60]. It should also be pointed out, that here we achieve these results by exploiting a resonant structure and more advanced fabrication processes [61]. Our simulations and measurements also demonstrate that silicon exhibits extraordinary time-varying capabilities that could potentially rival or even exceed those of transparent conductive oxides by leveraging resonant nonlinearities and plasma formation. While conductive oxides might have an edge in terms of transparency, any cavity environment can yield high transparency and large local fields, thus suggesting that silicon and other semiconductors could serve as viable alternative building blocks for time-varying metamaterials. Finally, we note that while our theoretical and experimental results show a remarkable degree of agreement, we are unable to find an apt comparison with other published work on the subject. For instance, high harmonic generation in solids and silicon is typically studied using time-dependent density functional theory, or TDDFT [62], [63], [64], or in environments where plasmonic antennas are placed on top of a silicon layer to enhance performance [65]. In references [62], [63], [64] odd harmonics are reported and featured, but no reference is made to even harmonic generation. Even harmonic generation is briefly mentioned in reference [65] but only to point out that it is not observed. Some speculation is offered to describe circumstances where it might appear, for example if inversion symmetry is broken for sufficiently large fields across electron-hole pairs. However, in all cases even harmonic generation is never quantified or described in terms of surfaces, intrinsic magnetism triggered by the Lorentz force, or convection. Therefore at least in this regard a direct comparison is not possible. In addition, while some TDDFT methods assume the interaction occurs in a single cell where the field is spatially uniform [62], other TDDFT approaches neglect propagation effects [64]. In our approach we do neither: fields are not uniform in cavity environments, since they are characterized by field localization. Therefore, a direct comparison with odd harmonic generation is currently unavailable.

## Supplementary Material

Supplementary Material Details

## References

[1] Smirnova D., Kivshar Y. S. (2016). Multipolar nonlinear nanophotonics. *Optica*.

[2] Butet J., Brevet P.-F., Martin O. J. F. (2015). Optical second harmonic generation in plasmonic nanostructures: from fundamental principles to advanced applications. *ACS Nano*.

[3] Krasnok A., Tymchenko M., Alù A. (2018). Nonlinear metasurfaces: a paradigm shift in nonlinear optics. *Mater. Today*.

[4] Shaltout A. M., Kildishev A. V., Shalaev V. M. (2016). Evolution of photonic metasurfaces: from static to dynamic. *JOSA B*.

[5] Carletti L. (2017). Controlling second-harmonic generation at the nanoscale with monolithic AlGaAs-on-AlOx antennas. *Nanotechnology*.

[6] Liu S. (2018). An all-dielectric metasurface as a broadband optical frequency mixer. *Nat. Commun.*.

[7] Shibanuma T., Grinblat G., Albella P., Maier S. A. (2017). Efficient third harmonic generation from metal–dielectric hybrid nanoantennas. *Nano Lett.*.

[8] Liu H. (2020). Beating absorption in solid-state high harmonics. *Commun. Phys.*.

[9] Shcherbakov M. R. (2021). Generation of even and odd high harmonics in resonant metasurfaces using single and multiple ultra-intense laser pulses. *Nat. Commun.*.

[10] Li J. (2020). Attosecond science based on high harmonic generation from gases and solids. *Nat. Commun.*.

[11] Zhong S., Liang Y., Wang S., Teng H., He X., Wei Z. (2022). High harmonic generation and application for photoemission spectroscopy in condensed matter. *Mater. Futures*.

[12] Peterka P. (2023). High harmonic generation in monolayer MoS2 controlled by resonant and near-resonant pulses on ultrashort time scales. *APL Photonics*.

[13] Rodríguez-Suné L. Retrieving linear and nonlinear optical dispersions of matter: combined experiment-numerical ellipsometry in silicon, gold and indium tin oxide. *Front. Photonics*.

[14] Centini M. (2008). Inhibition of linear absorption in opaque materials using phase-locked harmonic generation. *Phys. Rev. Lett.*.

[15] Roppo V., Foreman J. V., Akozbek N., Vincenti M. A., Scalora M. (2011). Third harmonic generation at 223 nm in the metallic regime of GaP. *Appl. Phys. Lett.*.

[16] Roppo V. (2009). Field localization and enhancement of phase-locked second- and third-order harmonic generation in absorbing semiconductor cavities. *Phys. Rev. A*.

[17] Roppo V. (2007). Role of phase matching in pulsed second-harmonic generation: walk-off and phase-locked twin pulses in negative-index media. *Phys. Rev. A*.

[18] Vincenti M. A., de Ceglia D., Roppo V., Scalora M. (2011). Harmonic generation in metallic, GaAs-filled nanocavities in the enhanced transmission regime at visible and UV wavelengths. *Opt. Express*.

[19] Scalora M. (2019). Resonant, broadband, and highly efficient optical frequency conversion in semiconductor nanowire gratings at visible and UV wavelengths. *JOSA B*.

[20] Rodríguez-Suné L., Trull J., Scalora M., Vilaseca R., Cojocaru C. (2019). Harmonic generation in the opaque region of GaAs: the role of the surface and magnetic nonlinearities. *Opt. Express*.

[21] Mukhopadhyay S. (2023). Three orders of magnitude enhancement of second and third harmonic generation in the visible and ultraviolet range from plasmonic gold nanogratings. *APL Photonics*.

[22] Tran R., Sly K., Conboy J. (2017). Applications of surface second harmonic generation in biological sensing. *Annu. Rev. Anal. Chem.*.

[23] Dutt A., Mohanty A., Gaeta A. L., Lipson M. (2024). Nonlinear and quantum photonics using integrated optical materials. *Nat. Rev. Mater.*.

[24] Gorlach A., Neufeld O., Rivera N., Cohen O., Kaminer I. (2020). The quantum-optical nature of high harmonic generation. *Nat. Commun.*.

[25] Grosse N., Bowen W., McKenzie K., Lam P. K. (2006). Harmonic entanglement with second-order nonlinearity. *Phys. Rev. Lett.*.

[26] Christov I., Murnane M., Kapteyn H. (1997). High-harmonic generation of attosecond pulses in the “single-cycle” regime. *Phys. Rev. Lett.*.

[27] Korobenko A. (2021). High-harmonic generation in metallic titanium nitride. *Nat. Commun.*.

[28] Tian W., Liang F., Lu D., Yu H., Zhang H. (2021). Highly efficient ultraviolet high-harmonic generation from epsilon-near-zero indium tin oxide films. *Photonics Res.*.

[29] Yang Y. (2019). High-harmonic generation from an epsilon-near-zero material. *Nat. Phys.*.

[30] Journigan T., Liu Y., Cabello C., Berrel S. N., Banerjee P., Chini M. (2024). High-harmonic generation in epitaxially grown zinc oxide films. *JOSA B*.

[31] Palik E. D. (1985). *Handbook of Optical Constants of Solids*.

[32] Toscano G. (2013). Nonlocal response in plasmonic waveguiding with extreme light confinement. *Nanophotonics*.

[33] Toscano G. (2015). Resonance shifts and spill-out effects in self-consistent hydrodynamic nanoplasmonics. *Nat. Commun.*.

[34] Ciracì C. (2012). Probing the ultimate limits of plasmonic enhancement. *Science*.

[35] Baghramyan H. M., Della Sala F., Ciracì C. (2021). Laplacian-level quantum hydrodynamic theory for plasmonics. *Phys. Rev. X*.

[36] De Luca F., Ciracì C. (2023). Impact of surface charge depletion on the free electron nonlinear response of heavily doped semiconductors. *Phys. Rev. Lett.*.

[37] Esteban R. (2015). A classical treatment of optical tunneling in plasmonic gaps: extending the quantum corrected model to practical situations. *Faraday Discuss.*.

[38] Haus J. W., de Ceglia D., Vincenti M. A., Scalora M. (2014). Quantum conductivity for metal–insulator–metal nanostructures. *JOSA B*.

[39] Sipe J., So V., Fukui M., Stegeman G. (1980). Analysis of second-harmonic generation at metal surfaces. *Phys. Rev. B*.

[40] Scalora M. (2018). Harmonic generation from metal-oxide and metal-metal boundaries. *Phys. Rev. A*.

[41] Scalora M. (2010). Second- and third-harmonic generation in metal-based structures. *Phys. Rev. A*.

[42] Bloembergen N., Pershan P. S. (1962). Light waves at the boundary of nonlinear media. *Phys. Rev.*.

[43] Maker P., Terhune R., Nisenoff M., Savage C. (1962). Effects of dispersion and focusing on the production of optical harmonics. *Phys. Rev. Lett.*.

[44] Fazio E. (2009). Complete spatial and temporal locking in phase-mismatched second-harmonic generation. *Opt. Express*.

[45] Gao J. (2021). Near-infrared to ultra-violet frequency conversion in chalcogenide metasurfaces. *Nat. Commun.*.

[46] Hallman K. A. (2023). Harmonic generation from silicon membranes at visible and ultraviolet wavelengths. *Opt. Express*.

[47] Tang W. (2024). Realizing high-efficiency third harmonic generation via accidental bound states in the continuum. *Opt. Lett.*.

[48] Tonkaev P. (2024). Even-order optical harmonics generated from centrosymmetric-material metasurfaces. *Phys. Rev. Res.*.

[49] Scalora M., Vincenti M. A., de Ceglia D., Grande M., Haus J. W. (2012). Raman scattering near metal nanostructures. *JOSA B*.

[50] Scalora M., Vincenti M. A., de Ceglia D., Grande M., Haus J. W. (2013). Spontaneous and stimulated Raman scattering near metal nanostructures in the ultrafast, high-intensity regime. *JOSA B*.

[51] Zalogina A. High-harmonic generation from a subwavelength dielectric resonator. *Sci. Adv.*.

[52] Ren S. Plasmon-Enhanced circular polarization high-harmonic generation from silicon. *Adv. Opt. Mater.*.

[53] Miller R. C. (1964). Optical second harmonic generation in piezoelectric crystals. *Appl. Phys. Lett.*.

[54] Boyd R. W. (2003). *Nonlinear Optics*.

[55] Burns W. K., Bloembergen N. (1971). Third-harmonic generation in absorbing media of cubic or isotropic symmetry. *Phys. Rev. B*.

[56] Centini M. (1999). Dispersive properties of finite, one-dimensional photonic band gap structures: applications to nonlinear quadratic interactions. *Phys. Rev. E*.

[57] Dachraoui H., Husinsky W. (2006). Thresholds of plasma formation in silicon identified by optimizing the ablation of lase pulse form. *Phys. Rev. Lett*..

[58] Scalora M. (2020). Electrodynamics of conductive oxides: intensity-dependent anisotropy, reconstruction of the effective dielectric constant, and harmonic generation. *Phys. Rev. A*.

[59] Besse V., Leblond H., Boudebs G. (2015). Fifth-order nonlinear susceptibility: effects of third order resonances in classical theory. *Phys. Rev. A*.

[60] Jaffray W. (2024). High-order nonlinear frequency conversion in transparent conducting oxide thin films. *Adv. Opt. Mater.*.

[61] Khurgin J. B. (2020). Adiabatic frequency shifting in epsilon-near-zero materials: the role of group velocity. *Optica*.

[62] Floss I. (2018). Ab initio multiscale simulation of high-order harmonic generation in solids. *Phys. Rev. A*.

[63] Freeman D., Kheifets A., Yamada S., Yamada A., Yabana K. (2022). High-order harmonic generation in semiconductors driven at near- and mid-infrared wavelengths. *Phys. Rev. B*.

[64] Tancogne-Dejean N., Mücke O. D., Kärtner F. X., Rubio A. Impact of the electronic band structure in high-harmonic generation spectra of solids. *Phys. Rev. Lett.*.

[65] Vampa G. (2017). Plasmon-enhanced high-harmonic generation from silicon. *Nat. Phys.*.

