# Supplementary material for: High-harmonic generation from subwavelength silicon films
Source: Nanophotonics. 2025 Jan 16;14(23):3927–38. doi: 10.1515/nanoph-2024-0468 (PMC12617717; doi:10.1515/nanoph-2024-0468)
Supplement: Supplementary file 1 — Supplementary Material Details [file j_nanoph-2024-0468_suppl_001.docx]

Supplemental Information:

High-harmonic generation from subwavelength silicon films

K. Hallman^1^, S. Stengel^2^, W. Jaffray^2^, F. Belli^2^, M. Ferrera^2^, M.A. Vincenti^3^, D. de Ceglia^3^, Y. Kivshar^4^, N. Akozbek^5^, S. Mukhopadhyay^6^, J. Trull^6^, C. Cojocaru^6^, and M. Scalora^7*^

^1^PeopleTec, Inc. 4901-I Corporate Dr., Huntsville, AL 35805, USA

^2^Institute of Photonics and Quantum Sciences Heriot-Watt University, SUPA Edinburgh,

EH14 4AS United Kingdom

^3^Department of Information Engineering – University of Brescia, 25123 Brescia, Italy

^4^Nonlinear Physics Centre, Australian National University, Canberra, ACT 2601, Australia

^5^US Army Space & Missile Defense Command, Tech Center, Redstone Arsenal, AL 35898 USA

^6^Department of Physics, Universitat Politècnica de Catalunya, 08222 Terrassa (Barcelona), Spain

^7^FCDD-AMT-MGR, DEVCOM AvMC, Charles M. Bowden Research Center, Redstone Arsenal, Alabama, 35898-5000, USA

*michael.scalora.civ@army.mil

**A. Experimental Setups**


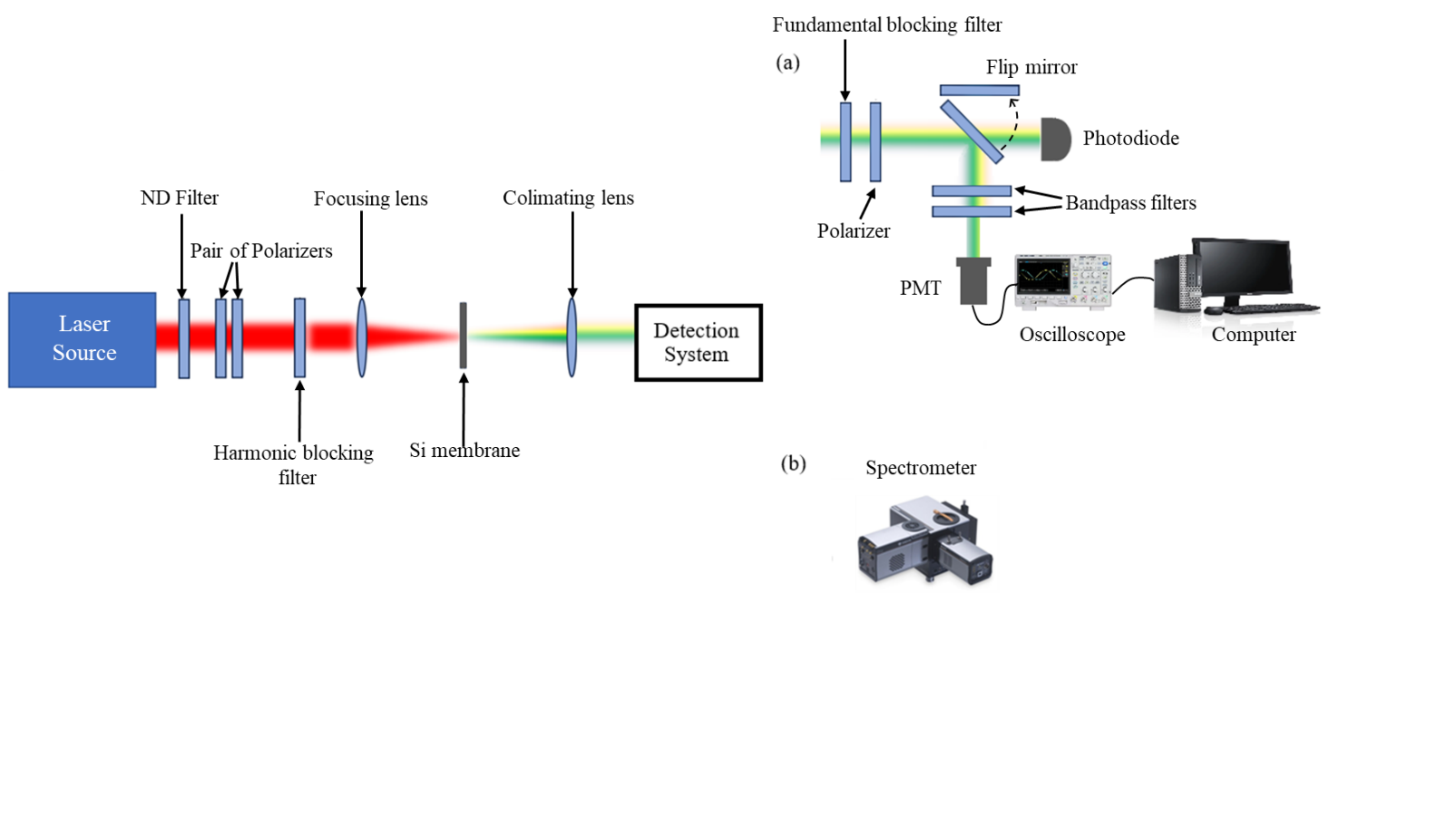


**Fig.S1:** Experimental setup used for measurement of conversion efficiencies as a function of incident pump power and angle for (a) peak power densities below 100GW/cm^2^; and (b) used for the measurement of the spectral response at peak power densities above 100GW/cm^2^.

Two experimental setups were deployed to measure different aspects of harmonic generation and are shown schematically in Fig.S1. The first setup (Fig.S1a) is used for peak power densities below 100GW/cm^2^ and is like the one outlined in Ref. [47] of the manuscript. This system is used to measure conversion efficiencies as a function of peak power density and angular dependence of the harmonic signals. It consists of an optical parametric amplifier (Coherent Opera Solo) pumped by an amplified Ti:Sapphire laser system (Coherent Astrella) and used as a source of femtosecond pulses with tunable wavelength and repetition rate. To prepare the pump beam, the OPA output was carefully filtered to allow through only the desired pump wavelength; neutral density filters and a pair of crossed polarizers adjusted the pump power, and a calcium fluoride lens with a long focal length was used to focus onto the samples. A second calcium fluoride lens collected the harmonic beam, and a polarizer was used to select the harmonic polarization. A mirror on a flip mount allowed the selection of either a calibrated silicon photodiode, or a more sensitive photomultiplier tube (PMT) to enable calibration of the responsivity of the full detection system including electronics used to collect the signal. The samples were mounted on a custom goniometer with six degrees of freedom, and the angle of incidence could be adjusted using a pair of rotational stages after aligning the sample to the center of rotation. For each point in the pump wavelength sweeps, the pump wavelength was changed manually, the laser energy adjusted to compensate for the wavelength dependent OPA efficiency, a separate responsivity value was used for the detection system, and a factor was used to compensate for the wavelength-dependent transmission (or reflection) of all optics. To measure higher order harmonics, especially the weaker even order harmonics, the intensity of the pump needed to be increased. Thermal loading of the samples was avoided by decreasing the repetition rate, sometimes as low as 2 Hz, and the integration time was increased.

Signal detection in the 200nm range was performed in a different lab with a different setup and laser system (shown in Fig.S1b). A Ti:Sapphire laser was used in conjunction with an OPA to generate 85fs pulses centered at 1475nm with a repetition rate of 10Hz. After the OPA, a visible filter was used to remove any residual pump with wavelengths shorter than 800nm. The beam is p-polarized, attenuated to the required power, and focused onto the film at normal incidence through a 200mm focal-length lens. This results in a laser spot size on the sample of 350µm (FWHM). After generating the higher harmonics, an uncoated CaF_2_ lens was used to collimate the beam. A grating was then used to spatially separate the harmonics, which are then sent through a slit and recorded by a photodiode (an integrated spectrometer was used to verify that the free space grating spectrometer was calibrated correctly). An input power of 1.5mW was used, which corresponds to a peak intensity of 3TW/cm^2^ on the sample. The sensitivity of the detection system was such that this peak power density was necessary to detect the 7^th^ harmonic at 210nm, which is the lower limit of our detection system, but not the lower limit of what is possible. The power RMS variation of our OPA’s output was measured to be below 1.5%. To further improve the certainty of our result under these conditions, we counteracted this power variation by monitoring the OPA’s energy output on a pulse-by-pulse basis and rejecting outliers. Beyond this, we utilized long integration times and many spectral averages to achieve consistent and repeatable results.

**B. 7^th^ harmonic generation vs incident wavelength.**

We simulated HHG across the spectrum shown in Fig.1b of the manuscript. The figure shows that it is possible to achieve relatively efficient 7^th^ harmonic generation below 100nm despite the presence of absorption at the pump wavelength, and despite the absence of local field amplification (see item **C.** below.) These results suggest that efficient harmonic generation below 100nm is possible by appropriately sizing a nanowire array made of materials like Si or GaP (see reference [19] in the manuscript).

**Fig.S2:** Simulated reflected (R) and transmitted (T) 7^th^ harmonic conversion efficiency vs incident pump wavelength in a range that spans the spectrum shown in Fig.1b., and incident peak power densities of 100GW/cm^2^. All things being equal, the resonance near 532nm is predicted to yield conversion efficiencies nearly one order of magnitude larger compared to the 1400nm resonance, despite partial absorption of the pump beam.

**C. Fabry-Perot (FP) etalon.**

In the simple FP etalon we are considering, the **H** and **E** fields delocalize, but the local electric field intensity is *not* amplified, notwithstanding the fact that the energy velocity of the pulse slows down to approximately *V_e_=0.2c*. This slow down corresponds to multiple passes that give rise to field maxima and minima inside the layer, with magnetic and electric field intensities tuned to 1400nm and 756nm as indicated in the figure.


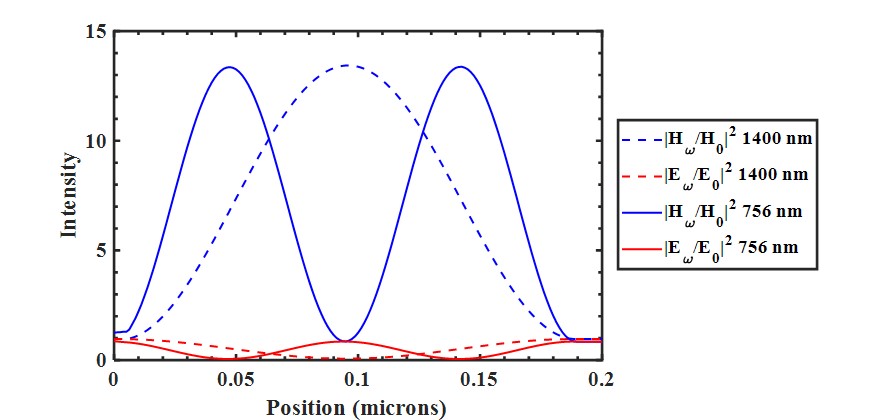


**Fig.S3:** Fabry-Perot modes at 1400nm and 756nm. The electric field intensity is not amplified. In each case, an H-field maximum coincides with an E-field minimum, leading to a reduction of the Poynting vector and decreased energy velocity.

**D. Integration Scheme in the Time Domain**

In what follows we report the most salient details of the method of integration, which is summarized from reference [RS1]. We consider a single Lorentz oscillator with a generic nonlinear term $\mathbf{P}^{NL}$. For simplicity we neglect the magnetic portion of the Lorentz force. The coupled Maxwell-Lorentz system may be written as follows:

$\nabla\times E=-\frac{1}{c}\frac{\partial H}{\partial t}; \nabla\times H=\frac{1}{c}\frac{\partial E}{\partial t}+\frac{4\pi}{c}\frac{\partial P}{\partial t}; \ddot{P}+\gamma\dot{P}+\omega_{0}^{2}P+P^{NL}=\frac{ne^{2}}{m}E\boldsymbol{.}$ (ES1)

The integration of Eqs.(ES1) in the time domain is carried out using the well-known split-step algorithm. The spatial derivatives of the fields are calculated using fast Fourier transforms (FFTs) using hundreds of thousands of plane waves in a two-dimensional spatial grid, while the polarization equation is integrated using a second-order accurate predictor-corrector method. The preservation of all spatial and temporal derivatives accounts for dynamical changes to the instantaneous phases and amplitudes of the fields. A TM-polarized **E** field has components in the y and z directions, the **H** field is polarized along the x-direction, and may be written as follows:

$E=\boldsymbol{j}E_{\hat{y}}+\boldsymbol{k}E_{\hat{z}}=\boldsymbol{j}\left( E_{TM\hat{y}}^{\omega}(r,t)e^{i(k\bullet r-\omega t)}+c.c. \right)+\boldsymbol{k}\left( E_{TM\hat{z}}^{\omega}(r,t)e^{i(k\bullet r-\omega t)}+c.c. \right)$**,** (ES2)

$H=\boldsymbol{i}H_{\hat{x}}=\boldsymbol{i}\left( H_{TM\hat{x}}^{\omega}(r,t)e^{i(k\bullet r-\omega t)}+c.c. \right)$**.**  (ES3)

Expanding Eqs.(ES1) on a two-dimensional spatial grid and time, fields, currents, and polarizations envelopes obey the following coupled equations of motion:

$$\frac{\partial H_{\hat{x}}}{\partial\tau}=i\beta\left( H_{\hat{x}}+E_{\hat{z}}\sin\theta_{i}+E_{\hat{y}}\cos\theta_{i} \right)-\frac{\partial E_{\hat{z}}}{\partial\hat{y}}+\frac{\partial E_{\hat{y}}}{\partial\hat{z}}$$

$$\frac{\partial E_{\hat{y}}}{\partial\tau}=i\beta\left( E_{\hat{y}}+H_{\hat{x}}\cos\theta_{i} \right)+\frac{\partial H_{\hat{x}}}{\partial\hat{z}}-4\pi\left( J_{\hat{y}}-i\beta P_{\hat{y}} \right)$$

$$\frac{\partial E_{\hat{z}}}{\partial\tau}=i\beta\left( E_{\hat{z}}+H_{\hat{x}}\sin\theta_{i} \right)-\frac{\partial H_{\hat{x}}}{\partial\hat{y}}-4\pi\left( J_{\hat{z}}-i\beta P_{\hat{z}} \right)$$

$$\frac{\partial J_{\hat{y}}}{\partial\tau}=\left( 2i\beta-\tilde{\gamma} \right)J_{\hat{y}}+\left( \beta^{2}+i\tilde{\gamma}\beta-\beta_{0}^{2} \right)P_{\hat{y}}+\frac{\pi\omega_{p}^{2}}{\omega_{r}^{2}}E_{\hat{y}}+P_{\hat{y}}^{NL}$$

$\frac{\partial J_{\hat{z}}}{\partial\tau}=\left( 2i\beta-\tilde{\gamma} \right)J_{\hat{z}}+\left( \beta^{2}+i\tilde{\gamma}\beta-\beta_{0}^{2} \right)P_{\hat{z}}+\frac{\pi\omega_{p}^{2}}{\omega_{r}^{2}}E_{\hat{z}}+P_{\hat{z}}^{NL}; \frac{\partial P_{\hat{y}}}{\partial\tau}=J_{\hat{y}}; \frac{\partial P_{\hat{z}}}{\partial\tau}=J_{\hat{z}}$**.** (ES4)

The electric and magnetic field equations may be put into a Schrödinger-like form, namely:

$$\frac{\partial H_{\hat{x}}}{\partial\tau}=V_{H_{\hat{x}}}H_{\hat{x}}-\frac{\partial E_{\hat{z}}}{\partial\hat{y}}+\frac{\partial E_{\hat{y}}}{\partial\hat{z}}$$

$$\frac{\partial E_{\hat{y}}}{\partial\tau}=V_{E_{\hat{y}}}E_{\hat{y}}+\frac{\partial H_{\hat{x}}}{\partial\hat{z}}$$

$\frac{\partial E_{\hat{z}}}{\partial\tau}=V_{E_{\hat{z}}}E_{\hat{z}}-\frac{\partial H_{\hat{x}}}{\partial\hat{y}}$**.** (ES5)

Eqs.(ES5) are solvable using the classic, split-step, beam propagation method adapted for the time domain [RS2]. The split-step algorithm usually calls for separation of free-space and material equations with differential equations that are first order in time. Eqs.(ES5) are already first order in time, have no approximations, can be immediately separated into free-space and material equations, and integrated in the time domain. The free-space propagator may be derived by setting the effective potentials equal to zero. Then, Eqs.(ES5) are Fourier transformed in space resulting in:

$$\frac{\partial\tilde{H}_{\hat{x}}}{\partial\tau}=-ik_{\hat{y}}\tilde{E}_{\hat{z}}+ik_{\hat{z}}\tilde{E}_{\hat{y}}$$

$$\frac{\partial\tilde{E}_{\hat{y}}}{\partial\tau}=ik_{\hat{z}}\tilde{H}_{\hat{x}}$$

$\frac{\partial\tilde{E}_{\hat{z}}}{\partial\tau}=-ik_{\hat{y}}\tilde{H}_{\hat{x}}$**.** (ES6)

Eqs.(ES6) may be integrated simultaneously using a midpoint trapezoidal method, so that:

$$\tilde{H}_{\hat{x}}(\delta\tau)=\tilde{H}_{\hat{x}}(0)-\frac{ik_{\hat{y}}\delta\tau}{2}\left( \tilde{E}_{\hat{z}}(0)+\tilde{E}_{\hat{z}}(\delta\tau) \right)+ \frac{ik_{\hat{z}}\delta\tau}{2}\left( \tilde{E}_{\hat{y}}(0)+\tilde{E}_{\hat{y}}(\delta\tau) \right)$$

$$\tilde{E}_{\hat{y}}(\delta\tau)=\tilde{E}_{\hat{y}}(0)+\frac{ik_{\hat{z}}\delta\tau}{2}\left( \tilde{H}_{\hat{x}}(0)+\tilde{H}_{\hat{x}}(\delta\tau) \right)$$

$\tilde{E}_{\hat{z}}(\delta\tau)=\tilde{E}_{\hat{z}}(0)-\frac{ik_{\hat{y}}\delta\tau}{2}\left( \tilde{H}_{\hat{x}}(0)+\tilde{H}_{\hat{x}}(\delta\tau) \right)$, (ES7)

which immediately yield:

$\tilde{H}_{\hat{x}}(\delta\tau)=\tilde{H}_{\hat{x}}(0)\frac{\left( 1-\frac{\left( k_{\hat{y}}^{2}+k_{\hat{z}}^{2} \right)\delta\tau^{2}}{4} \right)}{\left( 1+\frac{\left( k_{\hat{y}}^{2}+k_{\hat{z}}^{2} \right)\delta\tau^{2}}{4} \right)}+\frac{\left( ik_{\hat{z}}\tilde{E}_{\hat{y}}(0)-ik_{\hat{y}}\tilde{E}_{\hat{y}}(0) \right)\delta\tau}{\left( 1+\frac{\left( k_{\hat{y}}^{2}+k_{\hat{z}}^{2} \right)\delta\tau^{2}}{4} \right)}$. (ES8)

Eq.(ES8) is substituted back into the second and third of Eqs.(ES7) to extract the electric fields. All fields are then inverse Fourier transformed. The propagation step inside the medium is performed by integrating the material equations, also derived from Eqs.(ES4) and written in terms of generic envelope functions as:

$$\frac{\partial H_{\hat{x}}}{\partial\tau}=i\beta\left( H_{\hat{x}}+E_{\hat{z}}\sin\theta_{i}+E_{\hat{y}}\cos\theta_{i} \right)$$

$$\frac{\partial E_{\hat{y}}}{\partial\tau}=i\beta\left( E_{\hat{y}}+H_{\hat{x}}\cos\theta_{i} \right)-4\pi(J_{\hat{y}}-i\beta P_{\hat{y}})$$

$$\frac{\partial E_{\hat{z}}}{\partial\tau}=i\beta\left( E_{\hat{z}}+H_{\hat{x}}\sin\theta_{i} \right)-4\pi(J_{\hat{z}}-i\beta P_{\hat{z}})$$

$$\frac{\partial J_{\hat{y}}}{\partial\tau}=\left( 2i\beta-\tilde{\gamma} \right)J_{\hat{y}}+\left( \beta^{2}+i\tilde{\gamma}\beta-\beta_{0}^{2} \right)P_{\hat{y}}+\frac{\pi\omega_{p}^{2}}{\omega_{r}^{2}}E_{\hat{y}}+P_{\hat{y}}^{NL}$$

$$\frac{\partial J_{\hat{z}}}{\partial\tau}=\left( 2i\beta-\tilde{\gamma} \right)J_{\hat{z}}+\left( \beta^{2}+i\tilde{\gamma}\beta-\beta_{0}^{2} \right)P_{\hat{z}}+\frac{\pi\omega_{p}^{2}}{\omega_{r}^{2}}E_{\hat{z}}+P_{\hat{z}}^{NL}$$

$\frac{\partial P_{\hat{y}}}{\partial\tau}=J_{\hat{y}};\frac{\partial P_{\hat{z}}}{\partial\tau}=J_{\hat{z}}$**.** (ES9)

An approach similar to the solution of Eq.(ES7) may be employed to solve Eqs.(ES9). For instance, one may first obtain estimates of all fields currents, and polarizations at *τ =δτ  *(the prediction step), with an Euler method, using only their initial values at *τ=0*. Using these predictions, the solutions for the currents at *τ =δτ  *are immediate and second-order accurate, as follows:

$$J_{\hat{y},\hat{z}}(\delta\tau)=J_{\hat{y},\hat{z}}(0)\frac{\left( 1+\left( 2i\beta-\tilde{\gamma} \right)\frac{\delta\tau}{2}+\left( \beta^{2}+i\tilde{\gamma}\beta-\beta_{0}^{2} \right)\frac{\delta\tau^{2}}{4} \right)}{\left( 1-\left( 2i\beta-\tilde{\gamma} \right)\frac{\delta\tau}{2}-\left( \beta^{2}+i\tilde{\gamma}\beta-\beta_{0}^{2} \right)\frac{\delta\tau^{2}}{4} \right)}$$

$+\frac{\left( \beta^{2}+i\tilde{\gamma}\beta-\beta_{0}^{2} \right)P_{\hat{y},\hat{z}}(0)\delta\tau+\pi\frac{\omega_{P}^{2}}{\omega_{r}^{2}}\left( E_{\hat{y},\hat{z}}(0)+E_{P,\hat{y},\hat{z}}(\delta\tau) \right)\frac{\delta\tau}{2}+\left( P_{\hat{y},\hat{z}}^{NL}(0)+P_{P,\hat{y},\hat{z}}^{NL}(\delta\tau) \right)\frac{\delta\tau}{2}}{\left( 1-\left( 2i\beta-\tilde{\gamma} \right)\frac{\delta\tau}{2}-\left( \beta^{2}+i\tilde{\gamma}\beta-\beta_{0}^{2} \right)\frac{\delta\tau^{2}}{4} \right)}$. (ES10)

$E_{P,\hat{y},\hat{z}}(\delta\tau)$ and $P_{P,\hat{y},\hat{z}}^{NL}(\delta\tau)$ are first-order accurate, predicted estimates of the fields and nonlinear polarizations at time *τ =δτ*. Once the currents are known, the polarizations may be found using the usual, second order accurate trapezoidal rule:

$P_{\hat{y},\hat{z}}(\delta\tau)=P_{\hat{y},\hat{z}}(0)+\left( J_{\hat{y},\hat{z}}(\delta\tau)+J_{\hat{y},\hat{z}}(0) \right)\frac{\delta\tau}{2}$. (ES11)

In turn, knowledge of more accurate currents and polarizations at time $\delta\tau$ allows second-order accurate estimates of all electric and magnetic fields. The process is then repeated several times, although one or two cycles usually suffice for the results to converge (the correction step). The same procedure can be used for TE-polarized fields and negative index materials with a magnetically active medium. In our case the simulations are carried out on ordinary desktop computers, with typical execution times that span from a few minutes to several hours, depending on pulse width, incident angle, and spatial and temporal integration steps, which are 1.25nm and 4.125 x 10^-18^sec., respectively.

[RS1] M.Scalora et al. JOSA B **32**, 2129 (2015).

[RS2] M. Scalora and M. E. Crenshaw, Opt. Comm. **108**, 191 (1994).
